# Mercury in Red Knots (*Calidris canutus rufa*): Inter-Annual and Geographic Variation in Blood Mercury and Associated Toxicological Risk Along the Atlantic Flyway

**DOI:** 10.3390/toxics14090769

**Published:** 2026-08-28

**Authors:** Stephanie Feigin, Joanna Burger

**Affiliations:** 1 Wildlife Restoration Partnerships, 109 Market Lane, Greenwich, NJ 08323, USA; 2 Division of Life Sciences, Rutgers University, 604 Allison Road, Piscataway, NJ 08854, USA; burger@dls.rutgers.edu; 3 Environmental and Occupational Health Sciences Institute, Rutgers University, 170 Frelinghuysen Road, Piscataway, NJ 08854, USA

**Keywords:** mercury, red knot, Delaware Bay, horseshoe crab, migratory shorebirds, contaminants, bioindicator

## Abstract

Shorebird populations are declining sharply, and trace metal contaminants may compound the many migration-related threats they face by affecting feeding, migration, and reproductive success. Despite this risk to the federally threatened Red Knot (*Calidris canutus rufa*), blood mercury in Red Knots has not been examined outside Delaware Bay, and the associated toxicological risk has not been assessed across the annual cycle. We report blood mercury from four cohorts: spring 2024 and spring 2025 at Delaware Bay, fall 2024 at Avalon, New Jersey, and spring 2025 at Kiawah Island, South Carolina. Blood mercury was substantially higher in 2024 than in 2025 across both cohorts. In 2024, 38% of spring Delaware Bay birds and 62% of fall Avalon birds exceeded the 200 ng/g adverse sublethal risk threshold; no birds sampled in 2025 exceeded this level. No biological endpoints were measured, so this risk is inferred from published thresholds. Within single-day Delaware Bay flocks, body mass and mercury were positively related, an association consistent with bioaccumulation. Two cohorts had small samples, and site, season, and migratory stage are confounded in the design. Risk varies markedly among years, and a single sampling event is unlikely to characterize mercury exposure or its associated risk.

## 1. Introduction

Many shorebird species are experiencing severe global population declines, with some declining faster than almost any other avian group [1]. Although shorebirds face many threats throughout migration, contaminants may contribute to these declines by affecting feeding, migration, and reproductive success [1,2]. Understanding threats at stopover sites along migratory pathways helps managers and conservationists identify the locations that pose the greatest risk, particularly for long-distance migrants [3,4]. Identifying these threats requires baseline data, which depends on repeated sampling across multiple locations and time periods [5]. Most birds are exposed to trace metals, metalloids, and other contaminants through food and water. Aquatic birds are especially useful bioindicators of environmental contamination because they feed on aquatic organisms, a major route of exposure to pollutants, including point-source pollution and runoff [6]. As a result, foraging behavior and prey characteristics, including type, abundance, and availability, are key to understanding contaminant exposure and its effects on bird fitness.

Mercury (Hg) is a globally distributed neurotoxic trace metal that enters the environment through both natural processes, including volcanic activity, erosion, and ocean volatilization, and anthropogenic sources, primarily coal combustion and artisanal and small-scale gold mining (ASGM) [7,8]. Inorganic mercury deposited in estuarine systems is converted by anaerobic bacteria in sediments to methylmercury (MeHg), the highly toxic organic form that bioaccumulates and biomagnifies through food webs [9]. Since MeHg concentrates at each trophic level, species feeding at higher trophic positions can accumulate biologically significant amounts from prey that individually carry low concentrations. Blood mercury reflects recent dietary exposure, within days, and is therefore a sensitive and responsive indicator of food web mercury loading [10].

Red Knots (*Calidris canutus rufa*), a federally threatened shorebird species, make one of the longest avian migrations, traveling up to 15,000 km each year between Arctic Canadian breeding grounds and wintering sites in South America [11,12]. During northbound migration each May, they stop in Delaware Bay for a few weeks to refuel, nearly doubling their body mass before continuing north [13]. At this critical stopover, they feed almost exclusively on horseshoe crab (*Limulus polyphemus*) eggs [13,14,15], creating a direct link between mercury in crab eggs and mercury in shorebird blood [16]. Burger et al. [16] measured metals in horseshoe crab eggs and in the blood of shorebirds feeding on them at Delaware Bay and found a Kendall tau correlation close to 1.0 between the two. Since horseshoe crab egg mercury was not measured in the present study, the egg-to-blood pathway is treated as the motivating hypothesis rather than as a result. Moreover, previous trace metal studies in Delaware Bay documented significant increases in blood mercury between 2011–12 and 2019, with mean Red Knot blood Hg rising from 16.5 ± 3.1 ng/g to 92.1 ± 7.7 ng/g, an approximately six-fold increase over less than a decade [17,18]. These changes were discussed in the context of changes in U.S. mercury regulations and atmospheric deposition patterns [18].

Despite this established baseline and prior temporal comparison at Delaware Bay, several important gaps remain. First, blood mercury in *rufa* Red Knots has never been examined at geographic locations outside Delaware Bay. Since Red Knots use multiple stopover and wintering sites from South America to the Canadian Arctic, mercury exposure at Delaware Bay may not be representative of exposure across the full annual cycle. Second, no blood mercury data exist for Red Knots during southbound (post-breeding) migration, when birds returning from Arctic breeding grounds may carry contaminant burdens accumulated both at Delaware Bay and during the breeding season. Third, it is not known whether body mass, a critical variable in migratory physiology that differs systematically between northbound fueling birds and lean post-breeding southbound birds, is associated with blood mercury within or across cohorts. Understanding these relationships has direct conservation implications for Red Knots and other shorebirds. Mercury exposure may not be static across the annual cycle and may vary with location, season, diet, and year, so a risk assessment based on a single sampling event at one site may not represent the magnitude and pattern of exposure at other sites. For Red Knots, this is especially important because birds leave Delaware Bay for Arctic breeding grounds within days of spring stopover, carrying any mercury accumulated there into the most physiologically demanding stage of the year. Capturing the full picture therefore requires sampling across migratory sites, seasons, and years.

The goal of this study was to determine whether blood mercury concentrations in Red Knots differed (1) between years at the same location (Delaware Bay), (2) between Delaware Bay and other geographic locations, and (3) as a function of body mass. We sampled four cohorts of Red Knots in 2024 and 2025 at three locations in their migration including data during their northbound migration from South Carolina and Delaware Bay as well as data collected during their southbound migration from the New Jersey Atlantic Coast. We then compared our results to published data from 2011–12 and 2019 collected at Delaware Bay using identical methods. We present this as an exploratory assessment of spatial and temporal variation in blood mercury rather than a definitive test of geographic effects, because site, season, and migratory stage are confounded in our design. Since the new data span only two years, we treat comparisons with the earlier sampling as preliminary context rather than as evidence of a sustained temporal trend. With this study we tested the following null hypotheses:

(1) H_01_: There is no significant difference in blood mercury concentrations among sampling years (2011–12, 2019, 2024, and 2025) at Delaware Bay during spring northbound migration.
(2) H_02_: There is no significant difference in blood mercury concentrations between Red Knots sampled at Delaware Bay and those sampled at other geographic locations (Kiawah Island, SC; Avalon, NJ).
(3) H_03_: There is no significant relationship between body mass and blood mercury concentration in Red Knots. Since the Kiawah Island and Avalon cohorts also differ in season and migratory stage, H_2_ evaluates a combined geographic, seasonal, and directional contrast rather than the effect of location alone.

Any increase in mercury could indicate a potential health risk to Red Knots and, through the food chain, to other species [17,19]. Mercury is a well-known neurotoxin that has no essential biologic function and poses significant risks to vertebrates, including effects on reproduction, behavior, immune function, and survival [9,20]. The tight dietary linkage between horseshoe crab eggs and shorebird blood at Delaware Bay, combined with the inter-annual variability documented here, positions Red Knots as effective biological sentinels of mercury loading in this keystone estuarine food web.

## 2. Materials and Methods

### 2.1. Study Species and Capture Locations

This study focused on mercury levels in the blood of *rufa* Red Knots captured at three separate locations during their migration. Red Knots migrating through the Atlantic Flyway breed in the Canadian Arctic and overwinter in South America, primarily at Bahia Lomas (Tierra del Fuego, Chile), coastal Patagonia, and northern Brazil [11,12]. The *rufa* subspecies is federally listed as threatened in the United States [21] and endangered in Canada, primarily due to declining horseshoe crab egg availability at the Delaware Bay stopover [22]. Delaware Bay is one of the most important stopover sites for Red Knots during their northbound migration, with birds typically arriving from mid-May and using the site for 2–3 weeks before departing for breeding grounds [13,23].

**Figure 1:**
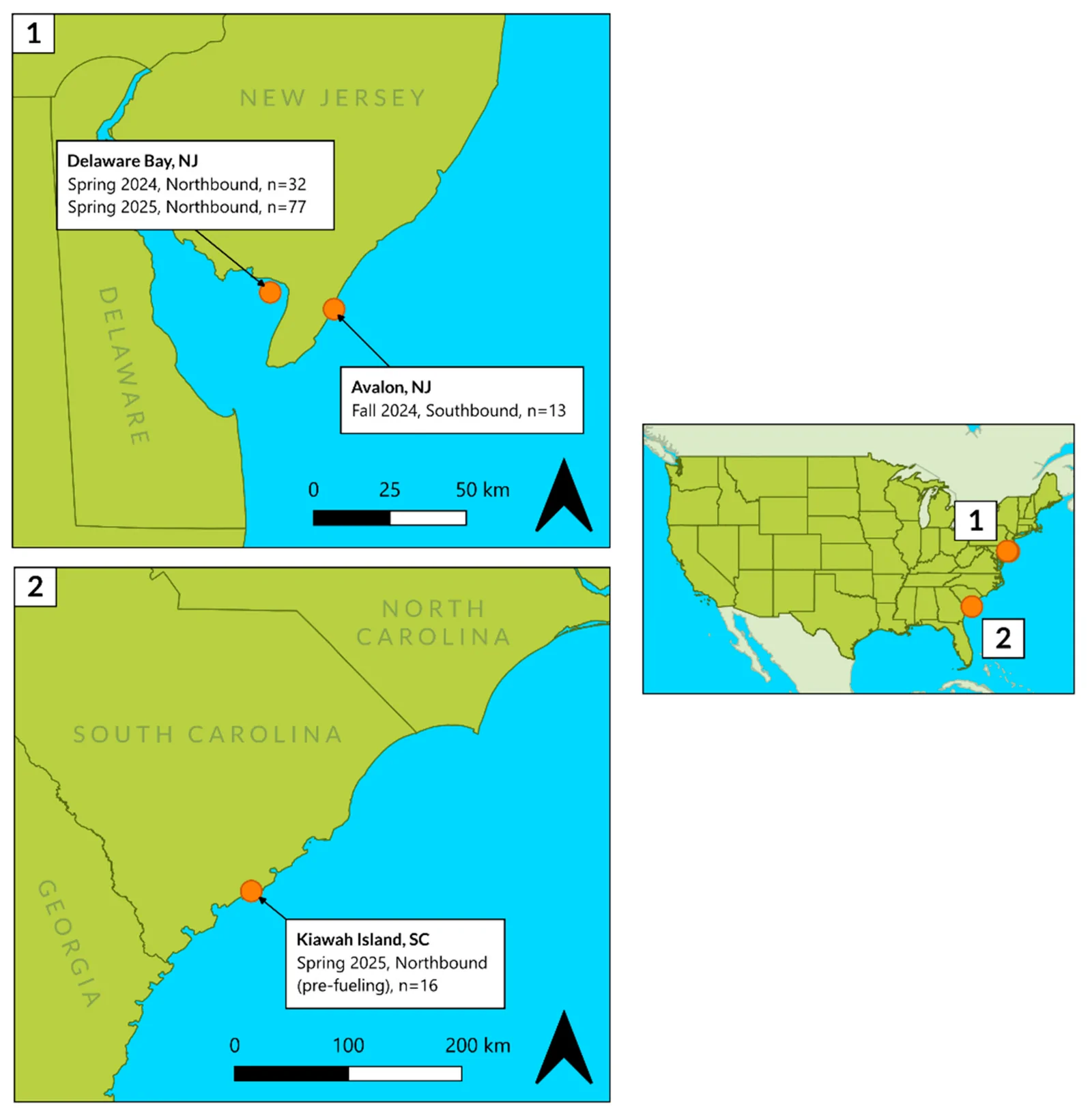
Map of project sampling locations including two years of sampling in Delaware Bay New Jersey, one year of sampling along the New Jersey Atlantic Coast and one year of sampling in South Carolina. Each site is labeled with the year, season, migratory direction and sample size of every cohort sampled there. Since each site was sampled in a different season and at a different stage of migration, site, season and migratory stage are confounded in this design. Contrasts between sites are therefore combined spatial, seasonal and directional contrasts rather than tests of location alone. Numbered boxes (1, 2) key the two detail panels to their positions on the locator inset at right. Orange circles mark sampling sites. Figure 1 was created with ArcGIS Pro v3.7, Esri, Redlands, CA, USA.

Four cohorts of Red Knots were sampled in 2024 and 2025 at three different sites in the northern hemisphere of their migration. These sites included Delaware Bay during northbound migration in 2024 and the NJ Atlantic Coast during their southbound migration in 2024, then both South Carolina in April and Delaware Bay in May during their northbound migration in 2025 (Figure 1). In May 2024, birds were sampled over 14–26 May at beaches on the upper Delaware Bay, New Jersey (*n* = 32). Capture locations were taken from the New Jersey master Red Knot banding database: 26 birds were caught at East Point and 6 at the southern end of Kimbles Beach. In October 2024, birds were sampled on the barrier island beaches at Avalon, New Jersey (NJ Atlantic coast), during southbound post-breeding migration (*n* = 13). In late March 2025, birds were sampled at Kiawah Island, South Carolina, a known northbound stopover and wintering site where Red Knots are present in spring (*n* = 16). Kiawah Island was included because it is a southeastern U.S. wintering and spring-stopover site used by *rufa* Red Knots earlier in the same northbound migration than Delaware Bay. Sampling there allowed a comparison of blood mercury before versus during the Delaware Bay fueling stopover. Here, in addition to potentially using South Carolina as a wintering site, some Knots migrate from other wintering sites and use South Carolina as a stopover before migrating to the Arctic, and some Knots stop in South Carolina then also stop in Delaware Bay before migrating to the Arctic. In May 2025, birds were sampled on the New Jersey shore of Delaware Bay during spring northbound fueling (*n* = 77), at the southern end of Kimbles Beach (*n* = 31, recorded locally as Bay Cove), at Moores Beach creek (*n* = 28) and at Norburys Landing (*n* = 18). All 2024 and 2025 data are new and reported here for the first time. Historical comparison data from Delaware Bay in 2011–12 (*n* = 30) and 2019 (*n* = 30) were drawn from Tsipoura et al. [17] and Burger and Feigin [18], respectively, which used identical methods, instruments, and technicians, ensuring direct comparability.

### 2.2. Blood Sample Collection

Shorebirds were captured by cannon nets at each site and placed in shaded holding cages protected from the sun, with all individuals held for less than 2 h prior to sampling, consistent with prior protocols in this series [16,17,18]. Approximately 100 μL of whole blood was drawn from the brachial vein using a 26 G ½ inch needle. Blood was drawn into heparinized capillary tubes and immediately frozen for transport. Body mass was recorded at the time of capture using an electronic balance (Ohaus 2200g OHAUS Corporation, Parsippany, NJ, USA). All methods were approved by the Rutgers University Institutional Animal Care and Use Committee (Protocol #92–036, renewed every three years) and were carried out under appropriate state and federal collecting permits.

### 2.3. Mercury Analysis

All samples were prepared and analyzed at the Environmental and Occupational Health Sciences Institute (EOHSI), Rutgers University. Separate laboratories were used for sample processing, digestion, and chemical analysis to prevent cross-contamination. All laboratory equipment and containers were washed and rinsed (10% HNO_3_ solution, deionized water) before use. Blood from each bird was individually homogenized and digested in 70% TraceMetal™ grade nitric acid (Fisher Chemical, Thermo Fisher Scientific, Waltham, MA, USA) in a microwave MDS 2000 CEM (CEM Corporation, Matthews, NC, USA). Total Hg was analyzed using a Perkin-Elmer FIMS-100 mercury analyzer (PerkinElmer Inc., Shelton, CT, USA) by cold vapor atomic absorption spectrophotometry, as in prior studies in this series [16,17,18]. The instrument detection limit was 0.02 ng/g for Hg. Quality assurance and quality control included certified reference material DORM-2 (Dogfish Muscle; National Research Council Canada, Ottawa, ON, Canada; certified Hg = 4470 ng/g) for every run, as well as spiked samples, procedural blanks, and replicate samples. Recovery rates ranged from 86 to 101% (coefficient of variation ≤ 10% on replicate spiked samples). The same instruments and technicians were used for all 2024 and 2025 analyses, ensuring direct comparability with the 2011–12 and 2019 data. All concentrations are expressed as ng/g wet weight (ppb) of whole blood.

### 2.4. Statistical Analysis

Non-parametric procedures were used throughout, which is consistent with prior studies in this series [16,17,18]. These procedures are followed because they are more conservative and best suited for datasets with small sample sizes [24]. Pairwise differences in blood mercury concentrations among cohorts were assessed using Mann–Whitney U tests. Since six pairwise comparisons were made among the four cohorts sampled in this study, *p*-values were adjusted using the Holm–Bonferroni method; both unadjusted and adjusted values are reported. Effect sizes are reported as the rank–biserial correlation with 95% confidence intervals from 10,000 bootstrap resamples, so that the magnitude and precision of each contrast can be judged independently of sample size. Confidence intervals on cohort geometric means were obtained the same way. The relationship between body mass and blood mercury was examined using Kendall tau rank correlations then calculated separately for each cohort and for all cohorts combined. We report arithmetic means ± standard errors (SE), medians, and geometric means throughout, as geometric means are preferred for log-normally distributed mercury data. Kruskal–Wallis tests were used where multiple group comparisons were made simultaneously, and statistical significance was set at *p* < 0.05 throughout. All analyses were performed in R version 4.3.3 (R Foundation for Statistical Computing, Vienna, Austria) [25] using RStudio 2026.05.0+218 (Posit Software, PBC, Boston, MA, USA), with the packages readxl, dplyr, tidyr and ggplot2.

## 3. Results

### 3.1. Body Mass by Cohort

Body mass differed significantly among cohorts, reflecting the distinct physiological states associated with migratory direction and fueling stage (Table 1). Spring Delaware Bay birds in 2025 were the heaviest (mean 169.9 ± 2.8 g, range 95–213 g), consistent with their refueling needs during the spring stopover before continuing their migration to their breeding grounds. Spring Kiawah Island birds, captured in late March before their continued migration to the Arctic or stopping over in Delaware Bay before the Arctic, were significantly lighter (mean 128.5 ± 1.8 g, range 112–140 g; Mann–Whitney *p* < 0.0001), reflecting a pre-fueling condition expected of birds recently arrived from wintering grounds. Fall southbound Avalon birds were the leanest of all cohorts (mean 118.5 ± 2.2 g, range 104–128 g), significantly lighter than both spring groups (all *p* < 0.001), consistent with their post-breeding post-flight from the Arctic. Mean body mass for spring 2024 Delaware Bay birds (140.8 ± 2.9 g) fell within the range of previously reported values [17,18], indicating that this cohort was also captured during active fueling, but it was significantly lower than that of the spring 2025 Delaware Bay cohort (Mann–Whitney *p* < 0.0001). The two spring Delaware Bay cohorts therefore differed by roughly 29 g in mean mass, which may reflect capture at an earlier point in the fueling period in 2024, a difference in fueling conditions between the two years, or both. In Table 1 we provide comparative data from 2011–12 and 2019 for later comparison.

### 3.2. Blood Mercury Concentrations by Cohort

Blood mercury concentrations differed markedly among cohorts (Table 2). Among the four cohorts, the 2024 fall Avalon southbound birds had the highest mean blood Hg (215.7 ± 20.9 ng/g ww; geometric mean 205.3 ng/g; range 112.7–422.3 ng/g), followed closely by the 2024 spring Delaware Bay birds (187.6 ± 17.8 ng/g; geometric mean 162.8 ng/g; range 58.7–410.7 ng/g). These two 2024 cohorts were statistically indistinguishable from each other (Mann–Whitney U = 153, *p* = 0.172), despite being captured at different locations five months apart and separated by a complete Arctic breeding season. In 2024, 78% of spring Delaware Bay birds (25 of 32) and 100% of fall Avalon birds (13 of 13) exceeded 100 ng/g Hg; 38% (12 of 32) and 62% (8 of 13), respectively, exceeded the adverse sublethal risk level (ASRL) of 200 ng/g established for sensitive birds and mammals [26].

In contrast, both 2025 cohorts showed substantially lower blood mercury. Spring Delaware Bay birds had a mean of 47.5 ± 3.7 ng/g (geometric mean 35.0 ng/g; range 1.2–138.2 ng/g), and spring Kiawah Island birds had a mean of 42.0 ± 9.1 ng/g (geometric mean 32.4 ng/g; range 11.6–145.1 ng/g). These two 2025 cohorts were not significantly different from each other (U = 710, *p* = 0.341). Only 8% of 2025 spring Delaware Bay birds (6 of 77) and 12% of Kiawah Island birds (2 of 16) exceeded 100 ng/g; no birds in either 2025 cohort exceeded the 200 ng/g ASRL.

All four pairwise comparisons between 2024 and 2025 cohorts were highly significant (each had *p* < 0.0001, and *p* < 0.0001 after Holm–Bonferroni adjustment), with rank–biserial effect sizes of 0.91 to 0.99. The two within-year comparisons (2024 spring DB vs. 2024 fall Avalon; 2025 spring DB vs. 2025 spring Kiawah, SC) were not significant in either case (both adjusted *p* = 0.345; Table 3). The confidence intervals on those two effect sizes are wide and span zero (−0.59 to +0.06 and −0.15 to +0.45), so they should not be read as evidence that the cohorts are equivalent. Statistical comparisons with the published 2011–12 and 2019 data are not reported here, as individual-level data are not available from those studies. Differences involving those years are therefore described qualitatively and are not statistical inferences.

### 3.3. Relationship Between Body Mass and Blood Mercury

**Figure 2:**
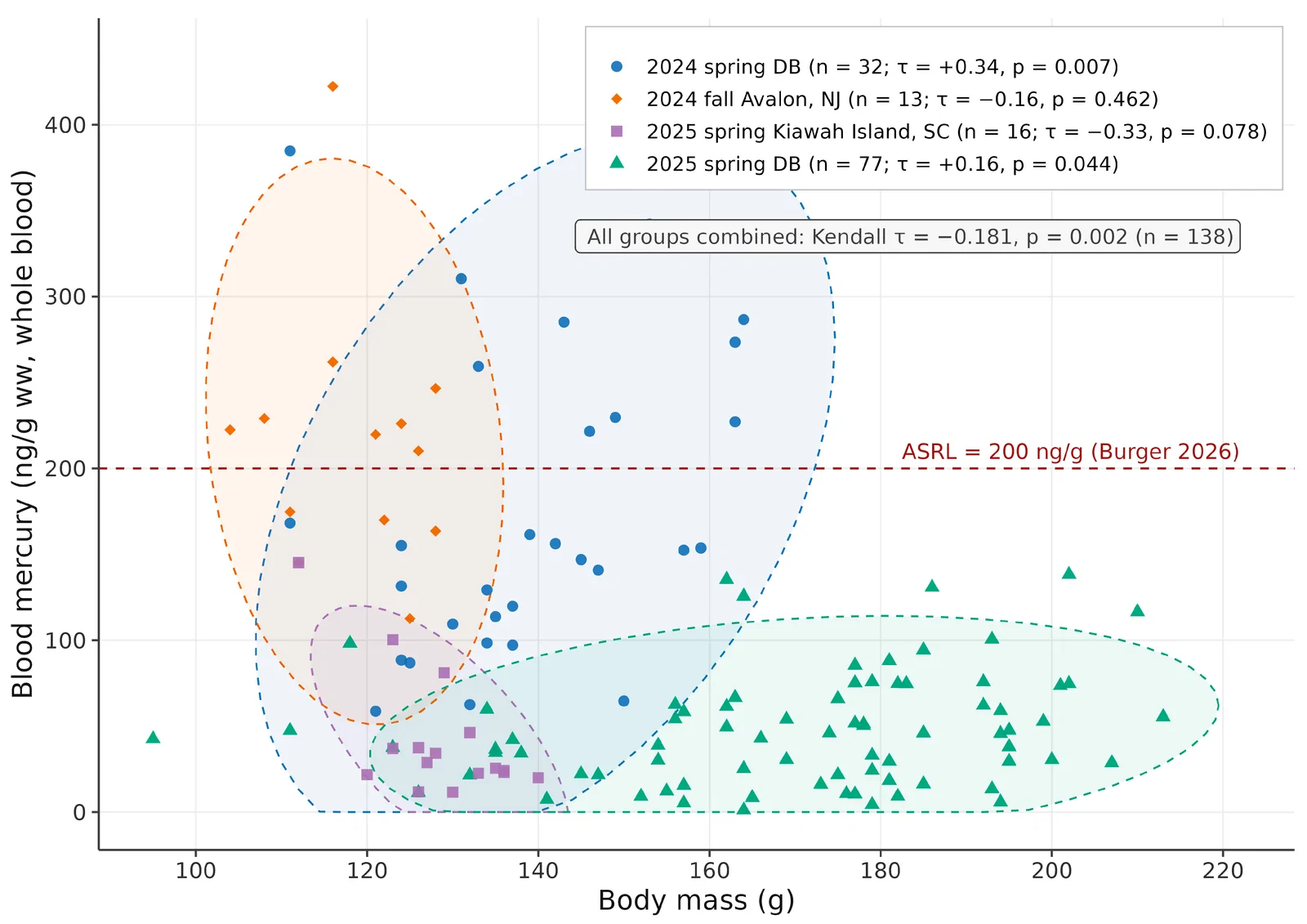
This figure shows the relationship between body mass (g) and blood mercury (ng/g ww, whole blood) in *rufa* Red Knots by cohort, 2024–2025 (*n* = 138; both measures were available for every bird). Each point represents an individual bird. Dashed ellipses show 1.5 SD confidence regions for each cohort, indicating the spread and orientation of each cohort’s mass–mercury distribution; ellipses are clipped at 0 ng/g. The dashed horizontal line marks the adverse sublethal risk level (ASRL = 200 ng/g; [26]). Cohort-level correlations are shown for reference only because capture beach and fueling stage are partly confounded within cohorts; the within-flock correlations in Table 4 provide the more reliable estimate of individual-level relationships.

The association between body mass and blood mercury varied among cohorts (Figure 2), but cohort-level correlations should be interpreted cautiously because in both spring Delaware Bay cohorts capture beach is partly confounded with timing within the fueling period. We therefore calculated correlations within each flock. Here, we defined flock as all birds captured at the same beach on the same day, where neither capture date nor capture site varies (Table 4). In the 2024 Delaware Bay cohort, heavier birds had higher blood mercury overall (Kendall τ = 0.34, *p* = 0.007), but this pattern disappeared after stratification. Within the 24-bird East Point flock captured on 14 May, there was no relationship (τ = 0.03, *p* = 0.82), and the cohort-level association was driven by six birds captured at a second beach twelve days later. The 2025 cohort showed a weaker correlation (τ = 0.16, *p* = 0.044), but the association appeared separately in each of its three flocks (τ = 0.27 to 0.32), yielding a pooled within-flock association of r = 0.33 (*p* = 0.001, *n* = 100).

No relationship was detected in the fall Avalon cohort (τ = −0.16, *p* = 0.46), where all 13 birds had high mercury levels regardless of body mass (112.7–422.3 ng/g), suggesting shared pre-arrival exposure rather than local accumulation during the stopover. No relationship was detected in the Kiawah Island cohort either (τ = −0.33, *p* = 0.078). Across all four cohorts combined, the correlation was weak but significantly negative (τ = −0.18, *p* = 0.002), driven by the lighter southbound Avalon birds with high blood mercury. Thus, the within-flock and pooled correlations ran in opposite directions, and the pooled value should not be interpreted as an individual-level pattern. Since arrival dates and residence times were not recorded for individual birds and capture date reflects only the date of blood sampling, we interpret the within-flock association as consistent with mercury bioaccumulation rather than as direct evidence of accumulation during the stopover. Testing that mechanism would require residence-time data and repeated measures from marked individuals.

## 4. Discussion

### 4.1. Year Effect and Why Repeated Sampling Across Time Is Essential

**Figure 3:**
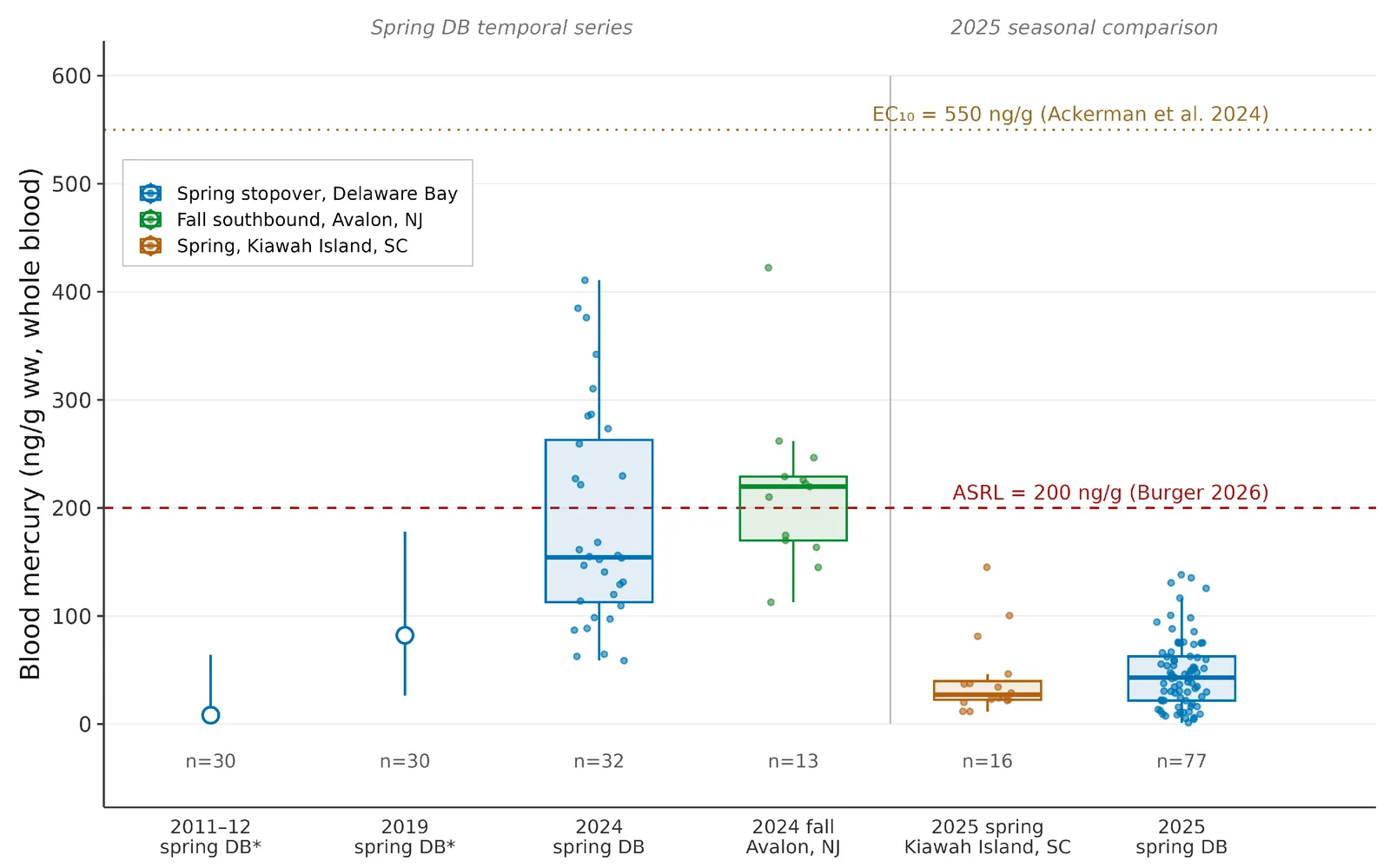
This figure shows blood mercury concentrations (ng/g ww, whole blood) in *rufa* Red Knots by sampling cohort, 2011–2025. For the four cohorts sampled in this study, boxes show the interquartile range, horizontal lines indicate medians, whiskers extend 1.5 × IQR, and jittered points represent individual birds. For the two published cohorts, individual-level data were unavailable. Therefore, these cohorts are shown only as reported geometric means (open circles) with minimum–maximum ranges, are not plotted as individual points, and are excluded from all statistical tests. The dashed red line indicates the adverse sublethal risk level (ASRL = 200 ng/g; [26]), and the dotted line indicates the EC_10_ for a 10% reduction in juvenile offspring production (550 ng/g; [27]). * Published data [17,18].

The most consequential finding of this study is the magnitude of year-to-year change in spring Delaware Bay blood mercury, a 75% decline between 2024 and 2025 at the same location and season, following a doubling between 2019 and 2024. Taken together, the four spring Delaware Bay time points span a 25-fold range from the 2011–12 geometric mean (8.2 ng/g) to the 2024 arithmetic mean (187.6 ng/g), yet within the dataset the full range from the lowest 2025 individual (1.2 ng/g) to the highest 2024 individual (422.3 ng/g) spans more than 350-fold. This inter-annual and individual-level variability suggests that a single sampling point captures mercury exposure only at that time. Since individual-level data are not available for the 2011–12 and 2019 cohorts, the changes described across these four time points are descriptive comparisons rather than a statistically demonstrated trend. In a migratory species whose diet, location, and food-web conditions shift across seasons and years, mercury exposure can vary substantially from year to year. Annual sampling is therefore essential for assessing yearly risk, because some years place more birds at mercury-related risk than others, adding another variable that may influence Red Knot populations. The full distribution of blood mercury across all sampling cohorts, including the historical 2011–12 and 2019 Delaware Bay data, is shown in Figure 3. One comparison is free of these confounds. Birds sampled at the southern end of Kimbles Beach on 26 May in consecutive years had a geometric mean blood mercury of 319 ng/g in 2024 and 38 ng/g in 2025. The 2024 sample is small (*n* = 5 against *n* = 31), but the two sets of values do not overlap at all, with the lowest 2024 bird at 227 ng/g exceeding the highest 2025 bird at 138 ng/g, and the same beach on the same calendar date in successive years isolates the year effect from site, season and migratory stage.

This finding supports Burger and Feigin’s [18] central argument that trace elements in shorebirds should be monitored regularly because blood concentrations reflect recent food-web conditions and can change rapidly. The present data extend that conclusion by showing that the direction of change can also reverse sharply within a single year. Long-term monitoring datasets, such as the one developed here by combining the 2024–2025 results with the 2011–12 and 2019 sampling events, are therefore valuable for characterizing mercury trajectories in Red Knots and potentially in other shorebirds using the same flyway.

### 4.2. The 2024 Anomaly: Observations and Potential Mechanisms

The high 2024 blood mercury values at Delaware Bay (mean 187.6 ng/g) were not predicted by wet atmospheric mercury deposition at the nearest long-term monitoring station, the NADP site NJ30 in New Brunswick, NJ. Using a 1–2 year estuarine food-web lag, the relevant deposition window for the 2024 spring birds was 2022–2023; during that period, mean annual Hg deposition was 7.43 μg/m^2^, the lowest value in the 15-year NJ30 record. Yet spring 2024 bird blood Hg was the highest observed in this dataset. By contrast, the 2017–2018 lagged window corresponding to the elevated 2019 bird values (mean 92.1 ng/g) had a higher mean deposition of 10.32 μg/m^2^, but even that level is insufficient, by linear scaling, to account for the 2024 concentrations. This decoupling suggests that at least one additional exposure pathway contributed in 2024. The three non-mutually exclusive mechanisms below are presented as hypotheses for future testing, ordered by the current level of support; none can be evaluated with the data collected here.

The most direct explanation for the elevated 2024 blood mercury values is a localized increase in horseshoe crab egg mercury at East Point or nearby upper-bay beaches. Egg mercury in Delaware Bay varies substantially across both years and beaches. Burger [26] reported mean egg mercury of 11.2 ng/g in 2019, 244 ng/g in 2021, and 61.2 ng/g in 2022, a more than 20-fold inter-annual difference, with beaches in the same year differing by as much as 60%. In that context, a single elevated year in one part of the bay would not be unusual. This hypothesis cannot be tested here because egg mercury was not measured at these beaches in 2023 or 2024. In May 2024, Juang et al. [28] reported egg mercury below detection at Reeds Beach. Reeds Beach, however, is in a different part of the bay, and given the spatial variation documented by Burger [26], that result neither supports nor rules out elevated egg mercury at East Point.

A second hypothesis is that storm-driven sediment resuspension in Delaware Bay may remobilize legacy mercury that was previously sequestered, making it bioavailable to the food web in episodic pulses. As storms become more frequent with climate change, they can release mercury from bay sediments independently of current atmospheric deposition. This mechanism could explain why mercury levels were anomalously high in 2024 without requiring elevated atmospheric deposition during 2022–2023 and why values returned quickly to lower levels in 2025 if similar storm forcing did not recur. Mercury may also have entered the bay through increased industrial runoff carried downstream by the Delaware River. Neither storm forcing nor sediment mercury flux was measured in this study.

Another hypothesis, carry-in of mercury acquired on the South American wintering grounds, is possible but the least likely. Artisanal and small-scale gold mining accounts for more than 83% of anthropogenic mercury emissions in South America [7], and bioaccumulation in the Tierra del Fuego coastal food web has been documented previously [29]. Therefore, wintering birds that feed on benthic invertebrates, including bivalves, amphipods, and marine worms that accumulate methylmercury from coastal sediments, may be exposed. However, two key observations argue against this explanation. First, gold prices and associated ASGM activity continued to rise through 2025, while spring blood mercury fell sharply, opposite the expected pattern. Second, blood mercury reflects only the preceding few days of exposure, making mercury acquired months earlier and thousands of kilometers south unlikely to remain detectable on arrival. We therefore do not pursue carry-in further. Therefore, the first two hypotheses are more likely, but distinguishing between the two would require simultaneous measurement of horseshoe crab egg mercury and Red Knot blood at the same beaches during the same weeks, which is beyond the scope of this study (Section 4.6).

### 4.3. Year-Wide Coherence: The 2024 and 2025 Annual Signals

An interesting feature of the dataset is the coherence of mercury levels within years across different sites and seasons. As noted above, site, season and migratory stage are confounded in this design, so the contrasts described in this section are combined spatial, seasonal and directional contrasts rather than tests of location alone. The two 2024 cohorts, spring Delaware Bay (mean 187.6 ng/g) and fall Avalon southbound (mean 215.7 ng/g), were statistically indistinguishable (*p* = 0.172) despite being captured at different locations, in different seasons, and in entirely different physiological states (spring migrants in active fueling at a mean of 140.8 g versus lean post-breeding birds at a mean of 118.5 g). Similarly, the two 2025 cohorts, spring Kiawah Island, SC (mean 42.0 ng/g), and spring Delaware Bay (mean 47.5 ng/g), were statistically indistinguishable (*p* = 0.341). The fact that within-year comparisons are not significant while all four cross-year comparisons are highly significant (each at *p* < 0.0001, and *p* < 0.0001 after Holm adjustment) is consistent with mercury exposure in this population being dominated by a year-wide signal rather than by site-specific or season-specific factors. That inference, however, rests on two years of new data from a small number of sites, and a longer series would be needed to confirm that a year effect predominates.

This annual coherence has an important implication for the fall Avalon birds specifically. If elevated blood Hg was driven solely by crab egg consumption at Delaware Bay in May 2024, we would expect the signal to diminish over the subsequent months as birds depurate mercury through feather molt during the breeding season. Instead, fall birds returned to New Jersey with mercury levels statistically equivalent to the spring values, suggesting that the elevated 2024 signal persisted through the Arctic breeding season and post-breeding migration or was reinforced by mercury from Arctic invertebrate prey during breeding. The absence of any significant weight–mercury relationship in the fall Avalon cohort, where all birds were lean regardless of their mercury level, further supports the interpretation that high mercury in these birds reflected a carried-in burden rather than ongoing accumulation at the Avalon site. Two caveats temper this interpretation. The Avalon and Kiawah samples are small (*n* = 13 and *n* = 16), and both cohorts of birds were not sampled on first arrival, so they could have acquired mercury at earlier stops, including known high-mercury staging areas such as Cape Cod, used by many geolocator-tracked Red Knots on southbound migration [30]. Moreover, since blood mercury accumulates only over the course of a few preceding days, these values reflect a recent but unknown, and possibly multi-site, exposure window rather than conditions at the capture site itself.

### 4.4. Mercury Levels Relative to Effect Thresholds and Conservation Implications

Mean blood mercury in both 2024 cohorts approached or exceeded the ASRL of 200 ng/g, above which adverse sublethal effects are considered likely for sensitive birds and mammals [26]. These comparisons are with published effect thresholds. No biological endpoints were measured in this study, so the risk described in this section is inferred rather than observed. In the fall Avalon cohort, 62% of individuals exceeded this threshold. These values are well below the EC_10_ for juvenile offspring production (550 ng/g; [27]) based on a comprehensive meta-analysis of 168 studies, suggesting that population-level reproductive impairment from blood mercury alone is not currently indicated at this level. However, there are still other important considerations.

First, the timing of exposure is critical. Spring Delaware Bay birds depart directly from the fueling stopover for Arctic breeding grounds, meaning any sublethal neurological, endocrine, or immune effects of the mercury accumulated during the stopover manifest during the most energetically demanding period of the annual cycle. Sub-threshold population-level effects on mean reproductive output may nonetheless be ecologically significant for a species already at unstable population levels. Second, the individual variation within cohorts is large. In 2024, the maximum individual value recorded was 422.3 ng/g in a fall Avalon bird, approaching the reproductive EC_10_ in the most exposed individuals. Third, mercury at sub-threshold levels can impair immune function and stress hormone response [20], potentially amplifying vulnerability to the multiple concurrent stressors Red Knots may already be facing, including declining horseshoe crab egg availability, habitat loss, and climate-driven mismatches at their Arctic breeding grounds [22]. Whether the mercury levels recorded in 2024 had any effect on survival or reproduction in that season cannot be determined from these data and remains to be tested directly.

The 2025 data are more reassuring; however, all 93 birds sampled in 2025 (across Delaware Bay and Kiawah Island) fell below the 200 ng/g ASRL, and only 8–12% exceeded 100 ng/g. Mean 2025 values are below the 2019 mean, suggesting a partial return toward pre-2019 conditions. Nevertheless, 2025 values remain approximately 2.9-fold above the 2011–12 baseline, indicating that a full recovery to pre-regulatory disruption levels has not occurred.

### 4.5. Red Knots as Sentinels of Estuarine Mercury Loading

Blood mercury in Red Knots at Delaware Bay has utility as a bioindicator of mercury loading in this estuarine system precisely because of the tight dietary linkage to horseshoe crab eggs and the rapid biological response time of blood (days to weeks). Burger and Feigin [18] demonstrated that As, Hg, and Se all increased significantly in shorebird blood between 2011–12 and 2019, consistent with changes in atmospheric deposition and horseshoe crab egg mercury levels. The present data begins to extend this sentinel function beyond Delaware Bay, although coverage outside the bay is limited to single sites in South Carolina and New Jersey, each sampled in a single season, so a flyway-wide characterization remains preliminary. The data from southbound birds at Avalon, NJ, and northbound birds at Kiawah Island, SC, provide windows into mercury exposure at migratory nodes that cannot be assessed at Delaware Bay alone. These first measurements away from Delaware Bay are a starting point towards a flyway-wide view of mercury exposure rather than a flyway-wide view in themselves. Red Knots stage and winter at a series of discrete nodes between Tierra del Fuego and the Canadian Arctic, and each carries its own contaminant signature. Extending blood sampling to these sites, anchored by the long-term Delaware Bay record, would allow for a deeper understanding of where birds acquire mercury, how that exposure shifts in space and time, and its larger potential threat to the birds throughout their migrations.

Horseshoe crab eggs and shorebird blood have been proposed as complementary bioindicators of the Delaware Bay food web [18,19]. The data presented here support this view. Changes in atmospheric deposition, sediment conditions, or local mercury inputs are likely to appear in bird blood within one migration stopover due to the rapid biological response time, making annual spring sampling at Delaware Bay a cost-effective monitoring tool. The inter-annual variability documented here, including the 2024 spike and the 2025 recovery, also highlights the value of sustained, repeated sampling for distinguishing real change from year-to-year variation. That applies both to the long-term single-site monitoring already established at Delaware Bay and to comparable effort at additional sites.

### 4.6. Limitations and Future Directions

Several limitations should be acknowledged. Most importantly, site, season, and migratory stage are confounded. Each site was sampled in a different season and at a different migration stage; therefore, site contrast does not isolate location alone. The fall Avalon (*n* = 13) and Kiawah Island (*n* = 16) samples are also small and unbalanced relative to the 2025 Delaware Bay sample (*n* = 77). Since these comparisons have limited statistical power, non-significant within-year differences should not be interpreted as biological equivalence; the corresponding effect-size confidence intervals are wide (Table 3). The new data also comes from small, single-time-point samples, limiting inference about temporal trends. The published 2011–12 and 2019 means provide useful context but lack individual-level data for this analysis.

The most important outstanding data gap is horseshoe crab egg mercury at East Point or nearby beaches in 2023 and 2024, which would allow the primary dietary pathway explanation for the 2024 anomaly to be directly evaluated. Sampling Red knots on their South American wintering grounds before northbound departure would allow the contribution of wintering-area carry-in to spring Delaware Bay blood mercury to be quantified for the first time. Finally, integrating multi-year blood sampling with concurrent crab egg sampling, NJ30 atmospheric deposition records, and storm activity data would permit formal path analysis of the multiple drivers of inter-annual variation in mercury exposure for this species. Simultaneous sampling of Red Knot blood and horseshoe crab eggs at the same beaches and in the same weeks would be the single most informative next step, since it is the only design that can test the dietary pathway directly.

## 5. Conclusions

Blood mercury in *rufa* Red Knots shows pronounced and non-linear inter-annual variation across single time-point assessments. At Delaware Bay, spring blood Hg measured 16.5, 92.1, 187.6 and 47.5 ng/g in four sampling years between 2011 and 2025, with 2025 values reflecting a substantial but incomplete recovery toward the historical baseline. Both 2024 cohorts, spring Delaware Bay and fall southbound Avalon, NJ, were statistically indistinguishable despite being separated by five months and a complete Arctic breeding season, indicating that the elevated 2024 signal was more likely a year-wide phenomenon than a Delaware Bay-specific or season-specific effect. Both 2025 cohorts, spring Delaware Bay and spring Kiawah Island, SC, were similarly indistinguishable from each other, confirming a system-wide low-mercury signal in 2025. A positive relationship between body mass and blood mercury was present within individual Delaware Bay flocks sampled on a single day in 2025, consistent with accumulation during the stopover, although the dietary source was not measured here and the relationship remains a statistical association. These findings are consistent with rejecting the three null hypotheses and indicate the value of multi-year, multi-site, multi-season sampling for characterizing contaminant exposure in long-distance migratory species. Shorebirds can serve as effective biological sentinels of mercury in estuarine food webs, but the inter-annual variability documented here argues strongly for sustained annual monitoring programs as a conservation priority for this federally threatened species. Moreover, these first measurements at sites outside of Delaware Bay are a potential starting point for tracking mercury and its impacts across the full migratory network.

## Figures and Tables

**Table 1:** Body mass (g) of rufa Red Knots by cohort, 2024–2025, with published comparison data. Published data: Tsipoura et al. [17] for 2011–12; Burger & Feigin [18] for 2019. All samples collected at Delaware Bay, NJ, during spring northbound stopover. The 2024 Delaware Bay cohort was sampled at East Point and at the southern end of Kimbles Beach and the 2025 cohort at Kimbles Beach south, Moores Beach creek and Norburys Landing. * Published comparison data; individual-level values were not available.

Group	*n*	Mean Body Mass ± SE (g)	Range (g)	Migratory Direction	Season/Location
**Published comparison**					
2011–12 spring Delaware Bay, NJ *	30	147.9 ± 6.1	98–204	Northbound	Spring, Delaware Bay, NJ, USA
2019 spring Delaware Bay, NJ *	30	152.8 ± 8.1	105–202	Northbound	Spring, Delaware Bay, NJ, USA
**This study**					
2024 spring Delaware Bay, NJ	32	140.8 ± 2.9	111–179	Northbound	Spring, Delaware Bay, NJ, USA
2024 fall Avalon, NJ (October)	13	118.5 ± 2.2	104–128	Southbound (post-breeding)	Fall, Avalon, NJ, USA
2025 spring Kiawah Island, SC (March)	16	128.5 ± 1.8	112–140	Northbound (pre-fueling)	Spring, Kiawah Island, SC, USA
2025 spring Delaware Bay, NJ	77	169.9 ± 2.8	95–213	Northbound (fueling)	Spring, Delaware Bay, NJ, USA

**Table 2:** Blood mercury concentrations (ng/g ww, whole blood) in *rufa* Red Knots by cohort, with comparison to published data. Arithmetic means are reported for comparability with the earlier studies in this series. Geometric means are included because blood mercury values are approximately log-normally distributed, making them a more appropriate measure of central tendency for these data. Confidence intervals on geometric means are percentile intervals from 10,000 bootstrap resamples. They cannot be computed for the published cohorts because individual-level data is not available. Both benchmark values are literature-based and were not measured as effect levels in this study: 100 ng/g is a commonly used lower screening benchmark, and 200 ng/g is the adverse sublethal risk level (ASRL) above which sublethal effects are considered likely for sensitive birds [26]. No individual in any cohort reached the EC10 of 550 ng/g, the concentration associated with a 10% reduction in juvenile offspring production [27]. * Published comparison data; individual-level values were not available.

Group	*n*	Mean Hg ± SE	Median Hg	Geomean Hg	Geomean 95% CI	Min	Max	n ≥ 100/≥200 ng/g
**Published (ng/g ww)**								
2011–12 spring Delaware Bay, NJ *	30	16.5 ± 3.1	~8.0	8.2	not available	0.3	64.1	—
2019 spring Delaware Bay, NJ *	30	92.1 ± 7.7	82.1	82.1	not available	26.3	178.0	—
**This study (ng/g ww)**								
2024 spring Delaware Bay, NJ	32	187.6 ± 17.8	154.3	162.8	134.5–195.6	58.7	410.7	25 (78%)/12 (38%)
2024 fall Avalon, NJ	13	215.7 ± 20.9	219.7	205.3	174.1–244.3	112.7	422.3	13 (100%)/8 (62%)
2025 spring Kiawah Island, SC	16	42.0 ± 9.1	27.2	32.4	23.4–45.8	11.6	145.1	2 (12%)/0 (0%)
2025 spring Delaware Bay, NJ	77	47.5 ± 3.7	43.0	35.0	28.5–42.6	1.2	138.2	6 (8%)/0 (0%)

**Table 3:** Pairwise two-tailed Mann–Whitney U comparisons of blood mercury among the four cohorts sampled in this study. NS = not significant; *** = *p* < 0.0001 before and after Holm–Bonferroni adjustment across the six comparisons. Effect size is reported as the rank–biserial correlation r, ranging from −1 to +1: r = +0.91 indicates that Group 1 exceeded Group 2 in 91% more bird-to-bird comparisons than the reverse, whereas r = 0 indicates complete overlap. Confidence intervals are percentile intervals from 10,000 bootstrap resamples. Published 2011–12 and 2019 cohorts were excluded because individual-level data were unavailable.

Group 1	Group 2	U Statistic	*p*-Value	Holm-Adjusted *p*	Effect Size r (95% CI)	Interpretation
2024 spring Delaware Bay, NJ	2024 fall Avalon, NJ	153	0.172	0.345	−0.26 (−0.59, +0.06)	NS—not different
2024 spring Delaware Bay, NJ	2025 spring Delaware Bay, NJ	2352	<0.0001	<0.0001	+0.91 (+0.83, +0.97)	*** Highly significant
2024 spring Delaware Bay, NJ	2025 spring Kiawah Island, SC	489	<0.0001	<0.0001	+0.91 (+0.77, +1.00)	*** Highly significant
2024 fall Avalon, NJ	2025 spring Delaware Bay, NJ	996	<0.0001	<0.0001	+0.99 (+0.96, +1.00)	*** Highly significant
2024 fall Avalon, NJ	2025 spring Kiawah Island, SC	206	<0.0001	<0.0001	+0.98 (+0.91, +1.00)	*** Highly significant
2025 spring Delaware Bay, NJ	2025 spring Kiawah Island, SC	710	0.341	0.345	+0.15 (−0.15, +0.45)	NS—not different

**Table 4:** Within-flock relationships between body mass and blood mercury. Flocks are defined as birds captured at the same beach on the same day. Only flocks with at least 10 birds are shown; together, these six flocks include 129 of the 138 birds sampled. Since capture date and site are constant within each flock, these correlations cannot be explained by differences among beaches or fueling stages. Geomean Hg is reported in ng/g ww. Combining the four Delaware Bay flocks with ranks centered within each flock gives a within-flock association of r = 0.33 (*p* = 0.001, *n* = 100).

Cohort	Beach	Date	*n*	Mean Mass (g)	Geomean Hg	Kendall τ (*p*)
2024 spring Delaware Bay, NJ	East Point	14 May 2024	24	136.0	145.7	+0.03 (0.82)
2024 fall Avalon, NJ	Avalon, NJ	2 October 2024	13	118.5	205.3	−0.16 (0.46)
2025 spring Kiawah Island, SC	Kiawah West	31 March 2025	16	128.5	32.4	−0.33 (0.078)
2025 spring Delaware Bay, NJ	Moores Beach creek	22 May 2025	28	159.2	41.8	+0.30 (0.027)
2025 spring Delaware Bay, NJ	Norburys Landing	24 May 2025	17	172.4	22.2	+0.32 (0.075)
2025 spring Delaware Bay, NJ	Kimbles Beach south	26 May 2025	31	177.8	37.9	+0.27 (0.035)

## Data Availability

The individual-level dataset and the R analysis script that reproduces every table, figure and statistic reported here are available from the corresponding author upon reasonable request.

## References

[1] Smith P.A., Smith A.C., Andres B., Francis C.M., Harrington B., Friis C., Morrison G., Paquet J., Winn B., Brown S. (2023). Accelerating declines of North American shorebirds signal the need for urgent conservation action. Ornithol. Appl..

[2] Ma Y., Choi C.-Y., Thomas A., Gibson L. (2022). Review of contaminant levels and effects in shorebirds: Knowledge gaps and conservation practices. Ecol. Environ. Saf..

[3] Webster M.S., Marra P.P., Haig S.M., Bensch S., Holmes R.T. (2002). Links between worlds: Unraveling migratory connectivity. Trends Ecol. Evol..

[4] Galbraith H., DesRochers D.W., Brown S., Reed J.M. (2014). Predicting vulnerabilities of North American shorebirds to climate change. PLoS ONE.

[5] Seewagen C.L. (2020). The threat of global mercury pollution to bird migration: Potential mechanisms and current evidence. Ecotoxicology.

[6] Burger J., Feigin S., Fojtik A., Dey A., Ng K. (2023). Bioaccumulation of some metals and metalloids in Laughing Gulls (Leucophaeus atricilla): Increases in mercury and decreases in selenium from 2019 to 2022/2023. Toxics.

[7] UNEP (2019). Global Mercury Assessment 2018.

[8] Streets D.G., Horowitz H.M., Lu Z., Levin L., Thackray C.P., Sunderland E.M. (2019). Global and regional trends in mercury emissions and concentrations, 2010–2015. Atmos. Environ..

[9] ATSDR (2013). Toxicological Profile for Mercury (Alkyl and Dialkyl Compounds).

[10] Perkins M., Ferguson L., Lanctot R.B., Stenhouse I.J., Kendall S., Brown S., Gates H.R., Hall J.O., Regan K., Evers D.C. (2016). Mercury exposure and risk in breeding and staging Alaskan shorebirds. Condor.

[11] Niles L.J., Sitters H.P., Dey A.D., Atkinson P.W., Baker A.J., Bennett K.A., Carmona R., Clark R.E., Clark N.A., Espoz C. (2008). Status of the red knot, Calidris canutus rufa, in the Western Hemisphere. Stud. Avian Biol..

[12] Niles L.J., Burger J., Porter R.R., Dey A.D., Koch S., Harrington B., Iaquinto K., Boarman M. (2012). Migration pathways, speeds and non-breeding areas used by northern hemisphere wintering Red Knots of the subspecies rufa. Wader Study Group Bull..

[13] Baker A.J., Gonzalez P.M., Piersma T., Niles L.J., de Lima I., Nascimento S., Atkinson P.W., Collins P., Clark N.A., Minton C.D.T. (2004). Rapid population decline in red knots: Fitness consequences of refuelling rates and late arrival in Delaware Bay. Proc. R. Soc. Lond. B.

[14] Tsipoura N., Burger J. (1999). Shorebird diet during spring migration stop-over on Delaware Bay. Condor.

[15] Duijns S., Niles L.J., Dey A., Aubry Y., Friis C., Koch S., Anderson A., Smith P.A. (2017). Body condition explains migratory performance of a long-distance migrant. Proc. R. Soc. B.

[16] Burger J., Tsipoura N., Gochfeld M. (2017). Metal levels in blood of three species of shorebirds during stopover on Delaware Bay reflect levels in their food, horseshoe crab eggs. Toxics.

[17] Tsipoura N., Burger J., Niles L., Dey A., Gochfeld M., Peck M., Mizrahi D. (2017). Metal levels in shorebird feathers and blood during migration through Delaware Bay. Arch. Environ. Contam. Toxicol..

[18] Burger J., Feigin S. (2025). Trace element contamination in three shorebird species migrating through Delaware Bay, New Jersey: Arsenic, mercury and selenium are increasing. Ecotoxicology.

[19] Burger J., Tsipoura N., Niles L., Dey A., Jeitner C., Gochfeld M. (2019). Heavy metals in biota in Delaware Bay, NJ: Developing a food web approach to contaminants. Toxics.

[20] Whitney M.C., Cristol D.A. (2017). Impacts of sublethal mercury exposure on birds: A detailed review. Rev. Environ. Contam. Toxicol..

[21] USFWS (2014). Threatened species status for the rufa red knot. Fed. Regist..

[22] Smith J.A.M., Dey A., Williams K., Diehl T., Feigin S., Niles L.J. (2022). Horseshoe crab egg availability for shorebirds in Delaware Bay: Dramatic reduction after unregulated horseshoe crab harvest and limited recovery after 20 years of management. Aquat. Conserv. Mar. Freshw. Ecosyst..

[23] Morrison R.I.G., Hobson K.A. (2004). Use of body stores in shorebirds after arrival in high Arctic breeding grounds. Auk.

[24] McDonald J.H. (2022). Handbook of Biological Statistics.

[25] R Core Team (2026). R: A Language and Environment for Statistical Computing.

[26] Burger J. (2026). Element levels in muscle and eggs of a keystone species, the Atlantic horseshoe crab (Limulus polyphemus): Risk to consumers that eat crabs depends upon tissue and route of exposure. J. Toxicol. Environ. Health A.

[27] Ackerman J.T., Peterson S.H., Herzog M.P., Yee J.L. (2024). Methylmercury effects on birds: A review, meta-analysis, and development of toxicity reference values for injury assessment based on tissue residues and diet. Environ. Toxicol. Chem..

[28] Juang Y.-P., Huang Y., Linder S., Keene M., Sheldon N., Burger J., Cernak T. (2025). Metabolomic profiles of Limulus polyphemus eggs. ChemRxiv.

[29] Fioramonti N.E., Ribeiro Guevara S., Becker Y.A., Riccialdelli L. (2022). Mercury transfer in coastal and oceanic food webs from the Southwest Atlantic Ocean. Mar. Pollut. Bull..

[30] Burger J., Porter R.R., Niles L.J. (2024). The migratory strategies of the Red Knots using the Western Atlantic Flyway as revealed by geolocators. Wader Study.

